# Intratumoral Gene Electrotransfer of Plasmid DNA Encoding shRNA against Melanoma Cell Adhesion Molecule Radiosensitizes Tumors by Antivascular Effects and Activation of an Immune Response

**DOI:** 10.3390/vaccines8010135

**Published:** 2020-03-19

**Authors:** Simona Kranjc Brezar, Valter Mrak, Masa Bosnjak, Monika Savarin, Gregor Sersa, Maja Cemazar

**Affiliations:** 1 Department of Experimental Oncology, Institute of Oncology Ljubljana, Zaloška 2, 1000 Ljubljana, Slovenia; skranjc@onko-i.si (S.K.B.); mbosnjak@onko-i.si (M.B.); msavarin@onko-i.si (M.S.); gsersa@onko-i.si (G.S.); 2 Bonifar d.o.o., Koprska ulica 108A, 1000 Ljubljana, Slovenia; valter.mrak@bonifar.si; 3 Faculty of Health Sciences, University of Ljubljana, Zdravstvena pot 5, 1000 Ljubljana, Slovenia; 4 Faculty of Health Sciences, University of Primorska, Polje 42, 6310 Izola, Slovenia

**Keywords:** melanoma cell adhesion molecule, siRNA, gene electrotransfer, irradiation, vascular targeted effect, immune response, mouse melanoma model, mouse carcinoma model

## Abstract

In this study, radiotherapy was combined with the gene electrotransfer (GET) of plasmid encoding shRNA against melanoma cell adhesion molecule (pMCAM) with dual action, which was a vascular-targeted effect mediated by the silencing of MCAM and an immunological effect mediated by the presence of plasmid DNA in the cytosol-activating DNA sensors. The effects and underlying mechanisms of therapy were evaluated in more immunogenic B16F10 melanoma and less immunogenic TS/A carcinoma. The silencing of MCAM potentiated the effect of irradiation (IR) in both tumor models. Combined therapy resulted in 81% complete responses (CR) in melanoma and 27% CR in carcinoma. Moreover, after the secondary challenge of cured mice, 59% of mice were resistant to challenge with melanoma cells, and none were resistant to carcinoma. Combined therapy reduced the number of blood vessels; induced hypoxia, apoptosis, and necrosis; and reduced cell proliferation in both tumor models. In addition, the significant increase of infiltrating immune cells was observed in both tumor models but more so in melanoma, where the expression of IL-12 and TNF-α was determined as well. Our results indicate that the combined therapy exerts both antiangiogenic and immune responses that contribute to the antitumor effect. However, tumor immunological status is crucial for a sufficient immune system contribution to the overall antitumor effect.

## 1. Introduction

Melanoma cell adhesion molecule (CD146/MCAM), which was originally discovered in melanoma cells [1], is a transmembrane glycoprotein of the immunoglobulin superfamily that is present in many tumors, such as melanoma, the pancreas, prostate, breast, kidney, ovarian cancer, mesothelioma, and small cell lung cancer [1,2,3,4,5,6,7]. The level of MCAM expression correlates with an aggressive and invasive phenotype of cancer and as such serves as a marker for poor prognosis [4,8,9]. MCAM is also expressed in different cells constituting the vessels, endothelial cells, pericytes, and smooth muscle cells [10,11,12] as well as in other types of cells, such as immune cells, mesenchymal stem cells, and many others [13,14]. It has been reported that the CD146 expressed in tumor cells is structurally and functionally different from that expressed in normal cells. Therefore, it would be reasonable to specifically target the MCAM expressed on tumor cells, which has already been performed by the development of antibodies recognizing only MCAM expressed in tumor cells [3,7]. Furthermore, the in vitro targeting MCAM using antibodies altered the biological properties of endothelial, melanoma, breast, ovarian, adenoid cystic carcinoma, and osteosarcoma cells, such as the proliferation or survival, migration, and invasion [3,15,16,17,18,19,20,21,22,23]. In particular, treating cells with antibodies against MCAM reduced proliferation for approximately 60%, migration for 75%, and tube formation in human umbilical vein endothelial cells (HUVEC), and invasion in melanoma and osteosarcoma cells, while the proliferation of melanoma, hepatocarcinoma, osteosarcoma, ovary, and cervix tumor cells was not affected [21,22,23,24]. In vivo, intratumoral treatment with antibodies against MCAM in melanoma, hepatocarcinoma, osteosarcoma, leiomyosarcoma, and pancreatic tumors reduced tumor growth and the formation of metastases in melanoma and osteosarcoma [21,23]. Specifically, peritumoral treatment of C81-61 melanoma xenograft for 46 days with a monoclonal antibody specific for tumor MCAM (TsCD146 mAb) significantly reduced tumor growth [3].

Another possibility is the direct local tumor targeting, either using siRNA molecules against MCAM or plasmid DNA encoding shRNA against MCAM [15,16,17,18,23,25]. These therapeutic approaches, utilizing siRNA or plasmid DNA encoding shRNA against MCAM delivered by electroporation (gene electrotransfer; GET), demonstrated effectiveness in vitro by the reduction in proliferation, survival, adhesion, migration, and invasion of the tumor and endothelial cells (antiangiogenic effect) [15,25]. Our recent in vivo study demonstrated the antitumor and antivascular effects of intratumoral GET with plasmid DNA-encoding shRNA against MCAM in melanoma B16F10, which resulted in significant tumor growth delay and 17% of tumor cures [15]. Furthermore, in that and in other studies from our group, we showed that the gene electrotransfer of control plasmid DNA, without the therapeutic gene, activated cytosolic DNA sensors that led to the release of inflammatory cytokines and consequently antitumor effectiveness [15,26,27,28,29]. Furthermore, in our previous studies, exploring the silencing of endoglin (CD105), which is involved in alternative angiogenic pathways in tumors, together with irradiation of tumors, we pointed out also the importance of immunogenicity of tumors to obtain a pronounced antitumor effect [26].

In the current study, we explore the potential of the combined treatment utilizing the irradiation of tumors with local GET with plasmid DNA-encoding shRNA against MCAM in two radioresistant tumor models. Furthermore, we wanted to compare the combined treatment in B16F10 melanoma, which is a more immunogenic tumor, with a high expression of MCAM to TS/A carcinoma, which is a less immunogenic tumor with a low expression of MCAM.

## 2. Materials and Methods

### 2.1. Cell Lines

The mouse mammary adenocarcinoma TS/A cells [30], less immunogenic [31,32,33,34] and mouse melanoma B16F10 more immunogenic cells (American Type Culture Collection, Manassas, Virginia, United States of America) [33,35] were cultured in advanced minimum essential medium (AMEM, Gibco, Thermo Fisher Scientific, Waltham, Massachusetts, United States of America) supplemented with 5% fetal bovine serum (FBS, Gibco), 10 mM/L L-glutamine (GlutaMAX, Gibco), 100 U/mL penicillin (Grünenthal, Aachen, Germany), and 50 mg/mL gentamicin (Krka, Novo mesto, Slovenia) in a 5% CO_2_ humidified incubator at 37 °C. Cells of at least 80% confluence were trypsinized using 0.25% trypsin/ethylenediaminetetraacetic acid in Hank’s buffer (Gibco), washed with AMEM with FBS, collected by centrifugation (470 g, 5 min, Heraeus, ThermoFisher) and used in in vitro and in vivo experiments.

### 2.2. Plasmids

In the experiments, two plasmids with constitutive CMV promoter were used, therapeutic plasmid DNA-encoding shRNA against MCAM (pMCAM; pENTR/U6 CD146) [15], and a control plasmid DNA-encoding shRNA with no homology to any gene in the mouse genome was used as a negative control (pControl; pENTR/U6 pControl) [26]. The plasmids were amplified in *Escherichia coli* (TOP10; Invitrogen) and isolated using a NoEndo Jetstar Endotoxin-free Mega/Giga Kit (Genomed, Löhne, Germany) according to the manufacturer’s protocol. The quantity of isolated plasmid DNA was determined by a spectrophotometer at 260 nm (Epoch Microplate Spectrophotometer, Take3 Micro-Volume Plate, BioTek, Bad Friedrichshall, Germany) and the quality was determined by measuring the ratio of absorbance at 260 nm/280 nm and by agarose gel electrophoresis. The working concentration of 4 mg/mL was prepared with endotoxin-free water.

### 2.3. In Vitro Gene Electrotransfer and Irradiation of Cells

Cells were trypsinized and washed two times in an ice-cold buffer (125 mmol/L sucrose; 10 mmol/L K_2_HPO_4_; 2.5 mmol/L KH_2_PO_4_; 2 mmol/L MgCl_2_ × 6H_2_0). Afterward, in GET, 44 μL of prepared cell suspension (25 × 10^6^ cells/mL) was mixed with 11 μl (1 µg/mL) of therapeutic or control plasmid DNA. Then, 50 μl of the resulting mixture (1 × 10^6^ cells) was pipetted between two stainless-steel parallel plate electrodes with a 2 mm gap in between. Eight square wave electric pulses (EP), with a voltage-to-distance ratio of 600 V/cm, pulse duration of 5 ms, and frequency of 1 Hz were generated by an electric pulse generator GT-01 (Faculty of Electrical Engineering, University of Ljubljana, Ljubljana, Slovenia). After the application of EP, the cells were incubated for 5 min with 100 μL of FBS and then plated in the particular medium. In addition to the GET group, there were also control groups: untreated cells (B16F10, or TS/A), cells treated with control plasmid DNA (pControl), cells treated with therapeutic plasmid DNA (pMCAM), and cells exposed to electric pulses without plasmid DNA (EP). One day after treatment, cells were trypsinized and collected by centrifugation, plated to Petri dishes (200–4000 cells/dish), and irradiated (IR) with single doses from 0 to 8 Gy at a dose rate of 2.16 Gy/min, using a Darpac 2000 X-ray unit (Gulmay Medical Ltd., Shepperton, United Kingdom) operating at 220 kV, 10 mA, with 1.8-mm aluminum filtration, as described previously [36]. Seven to 10 days after the irradiation, colonies were fixed and counted, and cell survival was determined. The experiment was repeated three times containing four repeats per group.

### 2.4. In Vitro Total RNA Extraction and Quantitative Reverse Transcription-Polymerase Chain Reaction (qRT-PCR) Analysis

To determine MCAM expression at the mRNA level in vitro 48 h after the GET of pMCAM in cells, total RNA extraction and qRT-PCR analysis were performed. Cells were trypsinized and then centrifuged. Total RNA was extracted from collected cells with a peqGOLD Total RNA kit (PEQLAB, VWR™, Life Science, Leuven, Belgium) according to the manufacturer’s instructions. The concentrations and purity of RNA were quantified spectrophotometrically using an Epoch Microplate Spectrophotometer (Take3TM Micro-Volume Plate, BioTek, Bad Friedrichshall, Germany). Total RNA (500 ng) was reversely transcribed into complementary DNA (cDNA) using a SuperScript VILO cDNA Synthesis Kit (Invitrogen, Thermo Fisher Scientific, Carlsbad, USA). Afterward, 10× diluted mixtures of transcribed cDNA were used as a template for the qRT-PCR using a TaqMan Gene Expression Master Mix (Applied Biosystems, Thermo Fisher Scientific) and TaqMan Gene Expression Assay (Applied Biosystems, Life Technologies). The TaqMan Gene Expression Assay was used for murine MCAM cDNA (Mm00522397_m1). As an internal positive control, TaqMan probes were used to amplify murine glyceraldehyde 3-phosphate dehydrogenase (GAPDH) (Mm99999915_g1), and as a negative control, a non-template control was used. The reaction was performed on Quant Studio 3 (Applied Biosystems, Life Technologies) and the results were analyzed with Quant Studio^®^ Design & Analysis Software v1.1 (Applied Biosystems, Life Technologies). The optimized thermal cycling conditions were as follows: the activation of uracil-DNA glycosylase (2 min at 50 °C), hot start activation of AmpliTaq Gold Enzyme (10 min at 95 °C), 40 cycles of denaturation (15 s at 95 °C), annealing, and extension (1 min at 60 °C). The MCAM and GAPDH mRNA expression levels in cells were presented as the threshold cycle value (Ct). The relative quantification was performed by comparison to the housekeeping gene GAPDH using the 2^−ΔΔCt^ method. To compare the expression of MCAM between control B16F10 and TS/a cells, the 2^−ΔCt^ method was used. The experiment was repeated four times with one sample for each group.

### 2.5. Animals

Female C57Bl/6JOlaHsd and BALB/cOlaHsd mice were purchased from Envigo RMS SrL (San Pietro al Natisone, Italy) and subjected to an adaptation period of 1 week. Mice were housed in specific pathogen-free conditions at a temperature of 20–24 °C, relative humidity 55 ± 10%, and a 12 h light/dark cycle. Food and water were provided ad libitum. All procedures were performed in compliance with the guidelines for animal experiments of the EU directive (2010/63/EU) and permission from the Veterinary Administration of the Ministry of Agriculture and the Environment of the Republic of Slovenia (permission no. U34401-1/2015/38). The experiment was repeated twice and the groups contained 6–12 animals per group. The exact number of animals per group used in the study is presented in Table 1.

### 2.6. In Vivo Gene Electrotransfer and Irradiation of Tumors

The subcutaneous tumors were induced on the back of mice by the injection of 100 µL of 1 × 10^6^ B16F10 cells in 0.9% NaCl in syngeneic C57BL/6JOlaHsd mice or 2 × 10^6^ TS/A cells in syngeneic BALB/cOlaHsd mice. In vivo experiments were performed as described previously [36]. When tumors reached 6 mm in the longest diameter, they were treated with an intratumoral injection of plasmid (12.5 μL) alone (pMCAM or pControl), an application of EP (8 square wave electric pulses with a voltage-to-distance ratio of 600 V/cm, a pulse duration of 5 ms, and a frequency 1 Hz), irradiation (IR), or a combination of all treatments (GET + IR). In GET, tumors were exposed to EP 10 min after the intratumoral injection of plasmid DNA. The EP were generated by the electric pulse generator ELECTRO CELL B10 (Betatech, L’Union, France) and delivered through two parallel stainless-steel electrodes with a 6 mm distance between them. After delivery of 4 pulses, electrodes were turned for 90° for delivery of 4 additional pulses to expose the whole tumor to GET. In total, GET was performed 3 times every second day (on days 0, 2, and 4).

One day after the first GET, tumors were irradiated. Mice were placed in special lead holders with the apertures for the local exposure of tumors. A single-dose of 15 Gy at a dose rate of 2.16 Gy/min was delivered by a Darpac 2000 X-ray unit (Gulmay Medical Ltd.) operating at 220 kV, 10 mA, with 1.8-mm aluminum filtration.

The antitumor effect was followed by measuring three orthogonal diameters (a, b, c) of tumors using a Vernier caliper every second day, and based on that, tumor volume was calculated according to the formula V = a × b × c × π/6. From the tumor volumes, arithmetic means for each group were calculated, and tumor growth curves were drawn. Error bars represent the standard error of the mean. The tumor doubling time was determined as the time at which the tumor doubled the volume from the initial day of the experiment. The tumor growth delay (GD) was calculated as the difference in the tumor doubling times of the therapeutic and control group. Tumors were followed until the tumor volume reached 350 mm^3^ (data were used for Kaplan–Meier survival curves). Animals with tumors in the regression were examined weekly for tumor presence for 100 days after the treatment. The animals were considered cured if they were tumor-free at day 100. Cured mice were challenged with a secondary subcutaneous injection of the tumor cells as described above in the right flank. Animals with no tumor growth at day 100 after the injection of tumor cells were considered as resistant to secondary challenge. Animal weight loss was monitored as a sign of systemic toxicity of the treatments. In addition, in irradiated animals, acute skin reactions in the irradiated field were monitored as described previously [37].

### 2.7. Histology

From each experimental group, three tumors were collected on day 6 from the beginning of the experiment to evaluate the histological properties of tumors. The tumors were fixed in zinc fixative for immunohistochemistry (IHC) (BD Biosciences, San Diego, CA, USA), embedded in paraffin blocks, and cut into the eight consecutive 2-μm thick sections. The first section was stained with hematoxylin and eosin (H&E) to estimate the percent of necrotic tumor area, the other seven sections were stained immunohistochemically to determine the percent of apoptosis, proliferation, hypoxia, immune cells (granzyme B), the interleukin-12 (IL-12) or tumor necrosis factor α (TNF-α)-positive cells, and the number of blood vessels.

The apoptosis was detected with antibodies against cleaved Caspase-3 (Ca-3, Cell signaling Technology, Danvers, Massachusetts, United States of America) at dilution 1:500 in TS/A and 1:1500 in B16F10, whereas for proliferation, antibodies against Ki-67 (clone SP6, Thermo Fisher Scientific) were used at a dilution of 1:1500 in TS/A and 1:1200 in B16F10. Antibodies against hypoxia inducible factor-1-α (ab2185, Abcam, Cambridge, Massachusetts, United States of America) at a dilution of 1:2000 in TS/A and 1:3500 in B16F10 were used to determine hypoxia, whereas antibodies against Granzyme B (ab4059, Abcam) were used for the staining of immune cells (cytotoxic T lymphocytes and natural killer cells) and were stained at a dilution of 1:1600 in TS/A and 1:1250 in B16F10. Interleukin-12 (IL-12) positive cells were determined using antibodies against IL-12 (ab203031, Abcam) at a dilution of 1:2000 in TS/A and 1:1000 dilution in B16F10. Tumor necrosis factor α (TNF-α)-positive cells were determined using antibodies against TNF-α (ab6671) at a dilution of 1:1600 in TS/A. Blood vessels were visualized with antibodies against CD31 (ab28364, Abcam) at a dilution of 1:1000 for TS/A and B16F10. Then, these primary antibodies were detected with a peroxidase-conjugated streptavidin-biotin secondary antibody (Rabbit specific HRP/DAB detection IHC kit, ab64261, Abcam) or a peroxidase-conjugated micro-polymer secondary antibody (Rabbit specific HRP/AEC IHC Detection Kit; Micro-polymer, ab236468, Abcam) and counterstained with hematoxylin as described previously [26]. With a BX-51 microscope (Olympus, Hamburg, Germany) under 10× magnification (numerical aperture 0.40), the whole area of the tumor H&E-stained section was captured by a DP72 CCD camera (Olympus) to analyze the necrotic area by two independent researchers and presented as the percent of the necrotic area on the tumor section. Images of immunohistochemically stained sections of at least five viable parts of each tumor sample were captured under 40× magnification (numerical aperture 0.85) (Olympus). Then, the images were analyzed by two independent researchers and presented as the percent of the cells on the acquired image (apoptosis, proliferation, hypoxia, immune cells, IL-12, and TNF-α-positive cells) or the number of blood vessels, as described previously [26,38]. The presence of IL-12 and TNFα-positive cells were determined as follows: (−), no cells in the field; (+), more than 1 cell in the field.

### 2.8. Statistical Analysis

SigmaPlot software (Systat Software, San Jose, California, United States of America) was used for statistical analyses. All data were tested for the normality of distribution with the Shapiro–Wilk test. The differences between the experimental groups were statistically evaluated by a one-way analysis of variance (one-way ANOVA) followed by a Holm–Sidak test for multiple comparisons. In the calculations of the difference between the doubling time of tumors, only tumors that did not regress completely were included. A P-value of less than 0.05 was considered to be statistically significant. Survival was estimated by the method of Kaplan–Meier, and survival curves were compared by the log-rank test.

## 3. Results

### 3.1. Radiosensitization of B16F10 Melanoma and TS/A Carcinoma Cells After GET of pMCAM In Vitro

**Figure 1:**
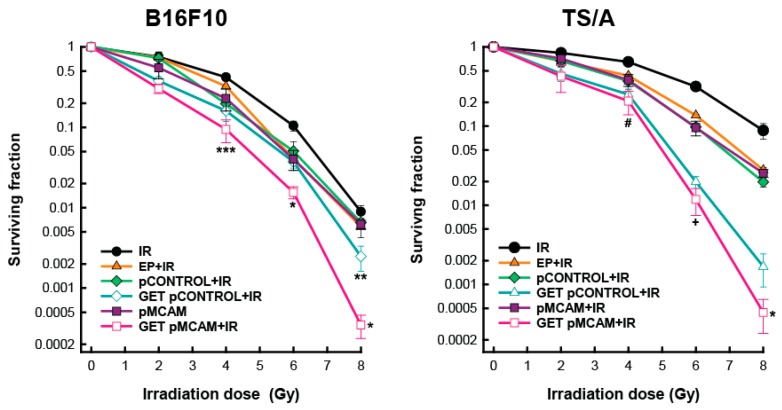
Survival of B16F10 melanoma and TS/A carcinoma cells after gene electrotransfer (GET) of plasmid DNA-encoding short hairpin RNA (shRNA) against melanoma cell adhesion molecule (pMCAM) and irradiation (IR). The data represent the mean and standard error of the mean (*n* = 12). EP: application of electric pulses; pControl: control plasmid; * *p* < 0.05 compared to all other groups; ** *p* < 0.05 between treatments for GET pControl+IR and GET pMCAM+IR; *** *p* < 0.05 compared to the IR and EP+IR groups; # *p* < 0.05 compared to the IR group; + *p* < 0.05 compared to all other groups except the GET pControl + IR group.

The survival of both cell lines was reduced after exposure to graded radiation doses. TS/A cells were more radioresistant than B16F10, resulting in an IC_90_ dose of 7.8 Gy compared to 6.1 Gy, respectively (Supplemetary Table 1). In both cell lines, radiosensitization was obtained after GET with either plasmid (therapeutic or control) at all radiation doses. Specifically, this non-specific radiosensitization was more pronounced in TS/A cells above 4 Gy. Furthermore, the radiosensitizing effect of MCAM silencing resulted in the enhancement factor of up to 1.72 in TS/A cells and to 1.57 in B16F10 cells. Enhancement factors of the combined treatment GET of control plasmid were lower in both cell lines (1.64 in TS/A and 1.30 in B16F10) and were comparable to that obtained by the combination of EP and IR (1.21 in TS/A cells and 1.24 in B16F10 cells) (Figure 1, Supplementary Table S1).

### 3.2. MCAM Silencing after GET of pMCAM in B16F10 Melanoma and TS/A Carcinoma Cells

**Figure 2:**
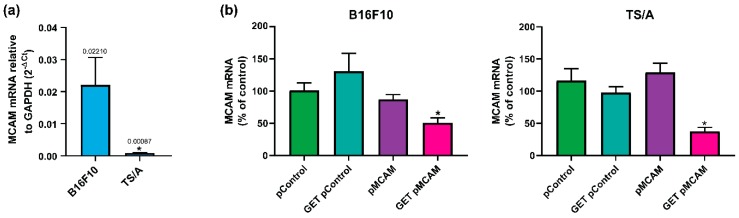
In vitro expression of MCAM mRNA level in untreated B16F10 melanoma and TS/A carcinoma cells (**a**) and reduced expression of MCAM mRNA level after silencing using gene electrotransfer (GET) in B16F10 melanoma and TS/A carcinoma cells (**b**) determined by qPCR analysis. (**a**) The data represent the mean and standard error of the mean (*n* = 4). The mRNA levels of MCAM in tumors were normalized to the mRNA level of the glyceraldehyde 3-phosphate dehydrogenase (GAPDH). * *p* < 0.05 compared to B16F10 melanoma. (**b**) The data represent the mean and standard error of the mean (*n* = 5). The mRNA levels of MCAM in cells of all experimental groups were normalized to the mRNA level of the untreated group. pMCAM: plasmid-encoding short hairpin RNA (shRNA) against melanoma cell adhesion molecule; pControl: control plasmid; * *p* < 0.05 compared to all other groups.

First, we determined the expression levels of pMCAM in both cell lines. The expression of MCAM was significantly 25 times higher in B16F10 melanoma cells compared to TS/A carcinoma cells (Ct value for MCAM expression in B16F10 was 24.7 and for TS/A, it was 30.9; Ct value for GAPDH expression in B16F10 was 19.2 and for TS/A, it was 20.7) (Figure 2a). Further, we determine the effectiveness of MCAM silencing 48 h after the GET of pMCAM. In both cell lines, only the GET of pMCAM significantly silenced the expression of MCAM; in B16F10 cells, it silenced the expression of MCAM by 44%, and in TS/A cells, it silenced the expression of MCAM by 63% (Figure 2b).

### 3.3. MCAM Radiosensitization of B16F10 Melanoma and TS/A Carcinoma After GET pMCAM In Vivo

**Figure 3:**
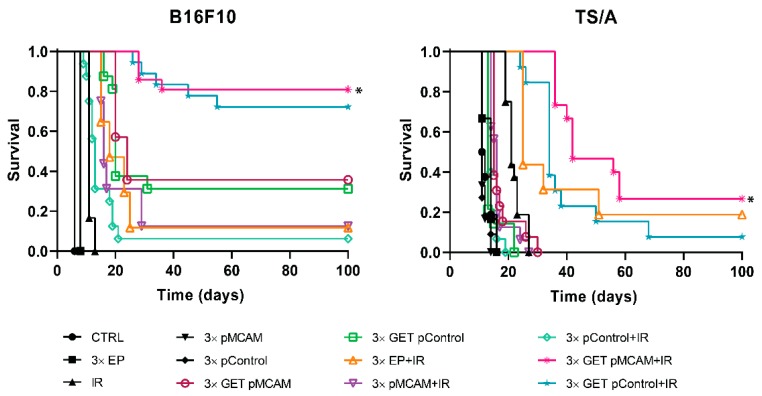
Kaplan–Meier survival curves of the mice bearing B16F10 melanoma or TS/A carcinoma tumors after treatment with gene electrotransfer (GET) of plasmid DNA-encoded shRNA against MCAM (pMCAM) and single-dose irradiation (IR, 15 Gy). The data represent the arithmetic mean and standard error of the mean, *n* = 9–21; * *p* < 0.05 significant to all other groups; CTRL: the control group; EP: application of electric pulses; pControl: intratumorally injection of control plasmid.

Monotherapies (application of electric pulses, EP; irradiation, IR; either of plasmids injection only) did not significantly affect tumor growth compared to the growth of control tumors in both tumor models (Table 1 and Table 2). Pertinent combined treatment—EP + IR, GET pMCAM, GET pControl, pMCAM + IR, and pControl + IR—resulted in prolonged tumor growth delay in both tumor models; however, it was not significantly prolonged compared to the control. In B16F10 melanoma, tumor cures were obtained in all these pertinent combined groups, ranging from 6% (pControl + IR) to 36% (GET pMCAM) (Table 1, Figure 3). On the contrary, in the TS/A tumor model, only the combination of EP and IR resulted in 19% tumor cures with up to 12 days of tumor growth delay (Table 2). This treatment result was significantly better compared to monotherapies or a treatment combination of pMCAM or pControl and IR (Table 2, Figure 3).

Combined treatment using GET pMCAM or pControl and IR in B16F10 melanoma resulted in significantly prolonged tumor growth delay compared to all other groups and in a high percentage of tumor cures (81% and 72%, respectively) (Table 1, Figure 2). In TS/A carcinoma, the number of tumor cures in this treatment combination was lower than in B16F10 melanoma. However, the combination with therapeutic shRNA molecule against MCAM resulted in a higher number of tumor cures (27%) compared to the combination with control plasmid pControl (8%) (Table 2, Figure 3).

**Figure 4:**
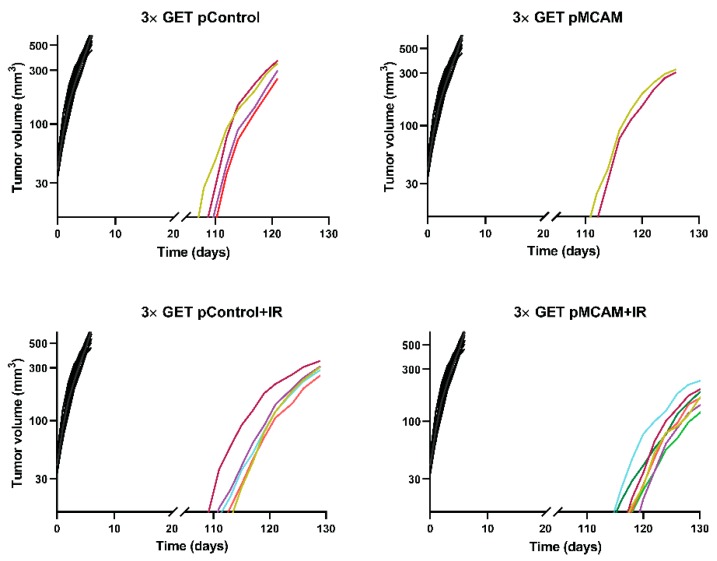
Tumor growth of individual B16F10 melanoma in mice that were re-challenged with tumors cells after being in complete response for 100 days. Animals that were cured for 100 days after the primary treatment were challenged with a secondary subcutaneous injection of the tumor cells (*n* = 2–7). The primary treatments that resulted in tumor cures are labeled in the graph title and were: GET pMCAM; GET pControl; GET pMCAM+IR; GET pControl+IR; all colored lines. The control naïve group (*n* = 18, black lines) represents the growth rate of individual tumors after the first inoculum, which received only tumor cells, the same as the re-challenged mice.

The mice with complete regression of the tumors were challenged with the injection of the tumor cells 100 days after the beginning of treatment to determine if the immune memory developed. In the B16F10 tumor model, there were more immunogenic tumors, and only mice that were treated with EP + IR were not resistant to secondary challenge, meaning that mice that received plasmid DNA developed immune memory to a certain degree. Namely, the percentage resistant to secondary challenge varied from 54% (GET pControl + IR) to 100% (pControl + IR and pMCAM + IR). In addition, the growth of these tumors was slower than the growth of the tumors in naive mice (Figure 4). In the TS/A tumor model, which is less immunogenic, all the challenged mice developed tumors.

### 3.4. Histologically Analyses

In obtained tumor tissues 6 days after the beginning of the therapy necrosis, apoptosis, hypoxia, proliferation, number of blood vessels, granzyme B-positive cells, and the presence of TNF-α and IL-12 were immunohistologically evaluated in relation to control, untreated tumors, and other pertinent controls. The data indicated a similar mode of action of combined treatments in both tumor models, but it was more pronounced in a more immunogenic B16F10 melanoma than in less immunogenic TS/A carcinoma.

**Figure 5:**
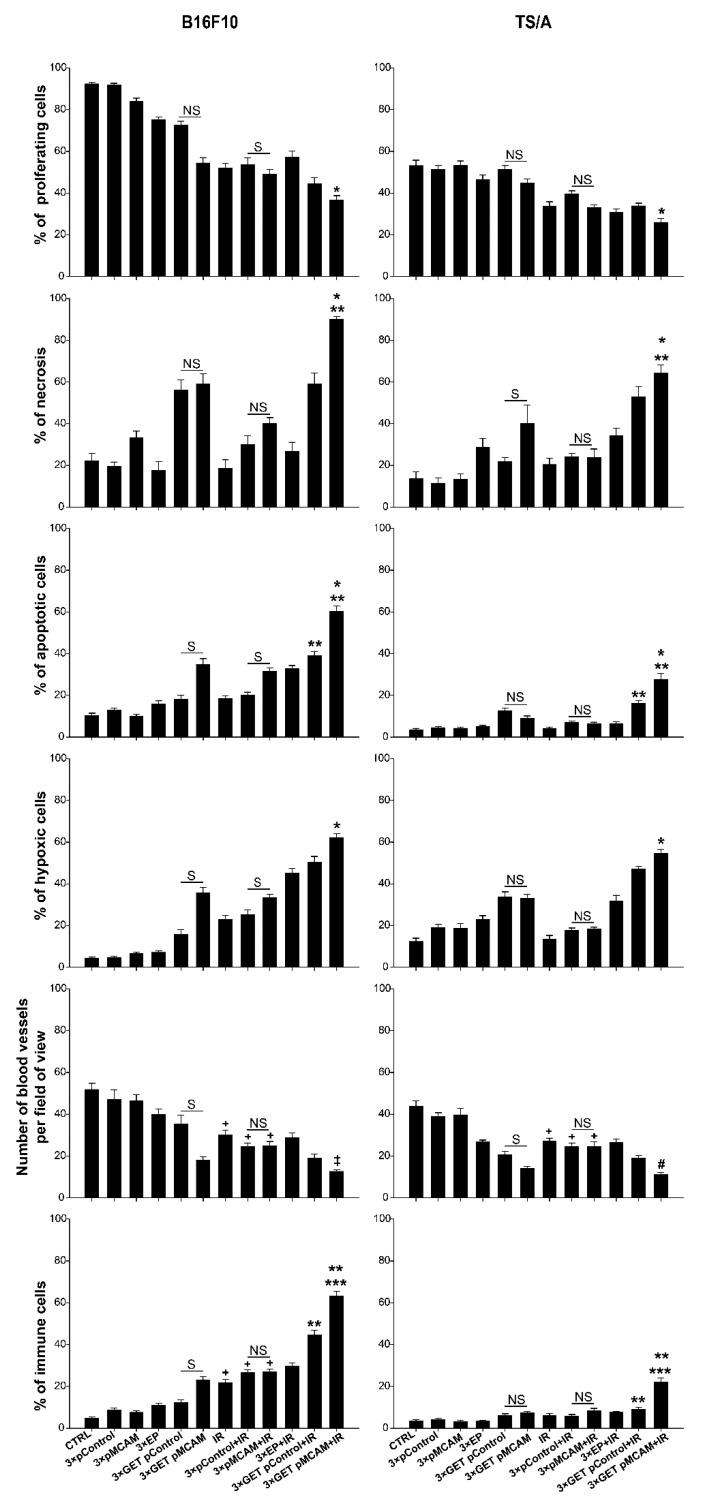
Histological analyses of B16F10 melanoma and TS/A carcinoma after gene electrotransfer (GET) with plasmid-encoding shRNA against MCAM (pMCAM) and single-dose irradiation (15 Gy). The data represent the arithmetic mean and standard error of the mean, *n* = 10 analyzed fields of view. *p* < 0.05 considered for statistically significant difference; NS: statistically non-significant difference between the therapeutic groups; S: statistically significant difference between the therapeutic groups; * compared to control and monotherapy groups; ** between tumor models; *** compared to all other groups; # compared to all groups except to GET pMCAM; ‡ compared to all groups except GET pMCAM and GET pControl + IR; + compared to the CTRL, pMCAM, and pControl groups.

**Figure 6:**
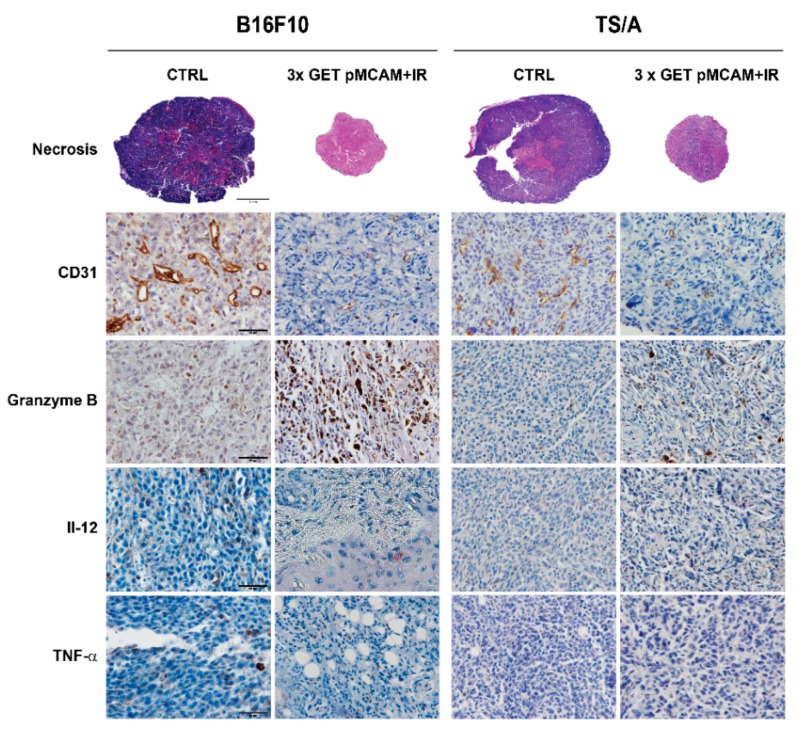
Histological sections of BF1610 melanoma nad TS/A carcinoma after gene electrotransfer (GET) with plasmid-encoding shRNA against MCAM (pMCAM) and single-dose irradiation (15 Gy) on day 6 after the beginning of the therapy. Brown staining represents CD31-positive tumor vessels or granzyme B-positive cells. Red staining represents IL-12 positive cells or TNF-α positive cells.

In general, untreated B16F10 tumors (93% of proliferating cells) were more proliferative than TS/A (53% of proliferative cells), confirming the tumor doubling time data. GET with either of the plasmids alone or combined with IR significantly decreased proliferation (Table 3 and Table 4, Figure 5) compared to the control tumors in both tumor models. The presence of necrosis in untreated tumors was approximately 20% in both tumor models. The percentage of tumor necrosis after treatments was higher in B16F10 than in TS/A, reflecting the tumor growth data, and it was significantly increased in tumors treated with GET pMCAM + IR. The same pattern was obtained for apoptosis and hypoxia, which were more pronounced in B16F10 tumors after the treatment reaching significance in the combined therapeutic group (GET pMCAM + IR). On the other hand, the number of blood vessels was decreased in both tumor models after the treatment with GET pMCAM + IR. Furthermore, the presence of granzyme-positive cells in these combined treatments was significantly increased, particularly in B16F10 melanoma. In addition, the presence of TNFα and IL-12 was only determined in B16F10 tumors treated with GET pMCAM + IR and GET pControl + IR, which correlates with the presence of granzyme B-positive cells (Table 3 and Table 4, Figure 5 and Figure 6). The expression of IL-12 and TNF-α in B16F10 melanoma was very low; a few cells were found in the GET pMCAM IR group (Figure 6).

## 4. Discussion

Preclinical studies have shown a silencing approach to target MCAM as valuable in cancer treatment. In vitro, silencing with plasmid DNA-encoding shRNA against MCAM reduced MCAM at mRNA and protein levels for different degrees in different cells, melanoma, endothelial cells, and breast cancer cells, with the highest silencing in melanoma and the lowest in endothelial cells [15,19]. In particular, in our previous study, in B16F10 melanoma cells, MCAM expression was reduced to a similar degree at mRNA and protein levels: 82% at the mRNA level and 69% at the protein level [15]. The antiangiogenic and antitumor properties after the silencing of MCAM were shown in studies in vitro and in vivo [15,19,23]. Reduced migration in HUVEC (70%) cells and in breast cancer (40%) after siRNA lipofection was determined [16,19]. In other studies, silencing MCAM with siRNA or plasmid DNA-encoding shRNA against MCAM reduced the proliferation in HUVEC for 40% in B16F1 melanoma cells for 79% and in B16F10 melanoma for 65% [15,16,17]. Our study confirmed the antiproliferative effect of MCAM silencing in tumor cells that have high-level (B16F10 melanoma) and low-level (TS/A carcinoma) expressions of MCAM, since silencing MCAM after GET reduced cell survival (the clonogenicity) by 85% in melanoma and 43% carcinoma. In addition, a significant reduction after GET of pMCAM in MCAM expression in melanoma and carcinoma was confirmed at the mRNA level.

Besides the antiproliferative and anti-migratory effects, MCAM silencing has also an antiangiogenic effect. Namely, in vitro inhibition of the ability to form capillary-like structures in 2H-11 cells after the GET of pMCAM and in HUVEC cells after antibody treatment against MCAM was demonstrated [15,23]. Further on, the inhibition of angiogenesis in a chicken chorioallantoic membrane assay in vivo with antibodies against MCAM was also demonstrated [23].

All these results suggested that the inhibition of MCAM may have an effect on endothelial cells and tumor cells in vivo and encouraged the researches to use the MCAM target in studies in vivo, not only in melanoma but also in osteosarcoma, pancreatic tumors, and prostate cancer bone metastases [15,21,23,39]. Silencing MCAM with plasmid DNA-encoding shRNA against MCAM after three consecutive GET in B16F10 melanoma was demonstrated in the study of Prosen et al. [15]. Significant tumor growth delays, as well as up to 25% cured tumors, were obtained after transfection either with magnetofection or GET, but the antitumor effect was more pronounced after GET [15].

GET can be used to target specific cells in tumors; in such cases, a tissue-specific promoter in plasmid DNA was constructed, i.e., a tissue-specific promoter of the endothelin-1 gene (pET-antiCD105, that was proved as effective as the plasmid with non-specific one [25]. In the current study due to the non-specific nature of the constitutive promoter, the silencing was obtained in tumor and endothelial cells. We performed therapy in tumors at 40 mm^3^, which was already well-vascularized; thus, the effects of silencing were observed in tumor and endothelial cells, which were demonstrated as reduced tumor cell proliferation and reduced tumor vasculature that induced hypoxia. Nevertheless, a high level of immune cell infiltration (compared to the control) in melanoma and significantly less in carcinoma indicated the indirect effect of therapy and activation of the immune response. Namely, in melanoma, which is an immunologically more responsive tumor, 36% of mice were cured and 60% of these mice were resistant to secondary challenge, while in carcinoma, which is a less immunogenic tumor model, no tumor cures were determined after the treatment with GET pMCAM. This was also confirmed with the treatment of GET using control plasmid (pControl), which is again more pronounced in melanoma; 31% of tumor cures were obtained after treatment and 20% of them were resistant to secondary challenge. Again, no tumor cures were obtained after treatment with GET of pControl in a carcinoma TS/A model. The antitumor effectiveness of nontherapeutic plasmids after GET was demonstrated also in many other studies in different tumor models, such as in melanomas, mammary carcinomas, fibrosarcomas, colorectal carcinomas, lung carcinomas, and pancreatic carcinomas [26,27,29,36,40,41,42,43,44,45,46,47]. A possible mechanism for this antitumor effect is the activation and upregulation of several cytosolic DNA sensors as a response to foreign DNA in the cells, acting as DAMPs (damage-associated molecular patterns) [28,29,48], inducing also the translation of several proinflammatory cytokines, such as type 1 interferons, TNF-α, and other cytokines [49]. This can enhance the non-specific killing of tumor cells by the innate immune system, enhance the presentation of tumor antigens, recruit cells of the adaptive immune system, and generate long-term memory against recurring tumor cells [50,51,52,53,54]. Specifically, in B16F10 melanoma, approximately 70% of animals were cured after GET with plasmid DNA (VR1255), and 70% of them were resistant to secondary challenge, suggesting the generation of an adaptive antitumor response [50]. However, this induction of immune response was more pronounced in the GET of therapeutic plasmids, as indicated in our study and others [26,36,37,42,51,55]. In a study silencing endoglin using the GET of a plasmid-encoding shRNA against endoglin under a tissue-specific promoter in the same melanoma tumor model B16F10, complete, long-term regression was found in 44% of tumors. In that study, 75% of these mice were resistant to secondary challenge [26]. Similar effects were demonstrated with GET of plasmid encoding IL-12 curing 47% of B16F10 tumors, of which 70% were resistant to secondary challenge [42], and in SA-1 sarcoma, where 50% cured animals were obtained, which were all resistant to secondary challenge [37]. Furthermore, by optimizing the electrotransfer protocol for GET of plasmid encoding IL-12 in the same tumor model (B16F10), long-term regression can be improved, resulting in around 80% cured animals, of which 50% were resistant to secondary challenge [51].

Another aspect that should be considered in the final antitumor effectiveness of GET is that electroporation can induce reactive oxygen production, mechanical stress, and heat, which can also trigger an immune response [56,57,58,59]. Taken all together, a direct effect on tumor cells and tumor vasculature, and indirect concomitant activation of immune response after GET contributed to the antitumor effect in our study. Furthermore, the contribution of EP to the radiosensitization of tumors has been already demonstrated (up to 27% cured animals) [26,36,37] and was confirmed also in our study, where around 12% cured B6F10 melanomas and 19% cured TS/A carcinomas were observed.

So far, the studies in vivo either inactivating MCAM by antibodies or the silencing of MCAM with plasmid DNA-encoding shRNA against MCAM have shown antitumor and antimetastatic effects, which are sum of the complex and complementary effects, such as the inhibition of proliferation of endothelial and tumor cells, migration, invasion, and antiangiogenic effect in endothelial cells [3,15,21,23,39]. Knowing that MCAM is involved in the development and progression of cancer disease through several pathways, it represents a good target in combined modalities (irradiation, chemotherapy) to treat cancer, with less possibility of resistance to treatment [9]. Since local irradiation induces a direct effect on tumor cells and tumor vasculature, it is used in many combined modalities [60]. Irradiation combined with the injection of either plasmid only moderately reduced tumor growth in both tumor models. Reduced tumor cell proliferation and increased levels of necrosis and apoptosis were determined histologically. In addition, the vascular effects of irradiation were determined as a reduced level of tumor vasculature in both tumor models, as indicated also in other studies [26,60,61]. This vascular effect was less evident compared to a group where MCAM was silenced with GET, but it was more pronounced in melanoma than in carcinoma. Furthermore, tumor-associated antigens released from death-irradiated cells, acting as DAMPs, can prime the immune system against the tumors [62,63,64,65]. Indeed, in our study, in melanoma tumor samples, significantly increased levels of immune cell infiltration after irradiation alone or combined with plasmids injection were compared to untreated tumors or treated with plasmid injection only, and we obtained tumor cures after combined treatment (12.5% using therapeutic and 6.3% using nontherapeutic plasmid) that were 100% resistant to secondary challenge, which indicated the immune system boosting. In addition, previously, we determined, using the same control plasmid as in the present study, an increased expression level of interferon β (more than 100 times compared to control untreated tumors) and TNFα (more than 9 times compared to control untreated tumors) after GET pControl, and tumor cures were obtained in 8% of cases [27]. Similar effects were demonstrated in the study of silencing endoglin using GET in the same B16F10 melanoma model, resulting in increased immune cell infiltration and 11% of cured tumors that were 100% resistant to secondary challenge [26]. Furthermore, in carcinoma, these effects were less evident, and a moderate increase of immune cell infiltration and no tumor cures were observed, as seen in the study silencing endoglin with GET [36]. Taken together, these results suggest the notion that the TS/A tumor model is less immunogenic than B16F10 melanoma [32,34,35,66].

Antivascular targeted gene therapies already proved pronounced tumor radiosensitization [26,31,37,67]. In this study, we demonstrated that MCAM silencing with GET effectively radiosensitizes tumors, both melanoma tumors that highly express MCAM and carcinoma tumors with a low expression of MCAM. First, a significant tumor growth delay and up to 81% melanoma and 27% of carcinoma-cured tumors were obtained. In addition, 59% of melanoma tumors were resistant to secondary challenge, while in carcinoma, none were resistant to secondary challenge. Furthermore, similar radiosensitization was obtained also with GET pControl resulting in 72% of tumor cures in melanoma and 8% in carcinoma. Again, the resistance to the secondary challenge was obtained in melanoma tumors only (54%). Histological analysis revealed the same mode of action in combined modality treatment with either plasmids GET (pMCAM or pControl), indicating direct antitumor and antivascular effects of the therapy; however, it was more pronounced in the samples where therapeutic plasmid was used for treatment. Furthermore, the immune cell infiltration was the highest in combined treatment with GET of either plasmids or irradiation, which was also demonstrated in our previous study [26]. Thus, it can be assumed that in combined treatment modality, the priming effect of irradiation complemented the GET of plasmid DNA, resulting in a pronounced boost of the immune system. However, this effect was present only in melanoma and not in carcinoma, where also the majority of cured animals became resistant to secondary challenge, indicating the development of long-term immune memory. In addition, TNFα and IL-12, which are both proinflammatory cytokines of mainly innate immunity, were present in B16F10 melanoma after the combined treatment and not in TS/A carcinoma. Since no resistance to the secondary challenge was obtained in other treatment combinations where only EP was used, we assumed that the presence of plasmid DNA is a prerequisite to obtain long-term immune memory. Furthermore, the outcome of the combined modality treatment response of tumors seemed to depend on tumor immunogenicity [26,36,66]. The result of the current study indicates the more pronounced response of melanoma to radiosensitization by silencing MCAM after GET than carcinoma, as significantly higher immune cell infiltration and resistance to the secondary challenge were determined. Thus, as already pointed out in other studies, the melanoma tumor model was presumed to be more immunogenic than carcinoma [26,36]. The appearance of vitiligo in the form of local discoloration of the fur at the treatment area was also supporting evidence, which was observed in all cured melanoma in C57Bl/6 animals (data not shown). Vitiligo, an autoimmune response against normal melanocyte triggered by ROS, was demonstrated in other studies as a positive marker for treatment response [26,38,68].

## 5. Conclusions

This study indicates a radiosensitization of two radioresistant tumor models, B16F10 melanoma and TS/A carcinoma, expressing high and low levels of MCAM, after silencing MCAM using the GET of pMCAM that was more pronounced in melanoma than in carcinoma. Radiosensitization seemed to be independent of the intrinsic sensitivity of cells to irradiation, since the higher enhancement factor of combined treatment was obtained in carcinoma cells in vitro. This effect of combined GET pMCAM + IR treatment can be mainly ascribed to a direct effect on tumor cells as well on tumor vasculature, since an increased level of apoptosis, reduced level of proliferation, and reduced tumor vasculature were determined. Furthermore, this combined treatment acted also indirectly, which was demonstrated by the GET of nontherapeutic plasmid that activated immune cell infiltration in both tumor models, but long-term immune memory was obtained in melanoma only, resulting in a 20% rejection to secondary challenge. The irradiation of tumors mainly affected tumor cells, thus promoting the release of tumor-associated antigens and tumor vasculature to some extent. When irradiation was used in treatment modalities, the immune system was boosted, which was determined by an increase in immune cell infiltration, and again, it was more pronounced in melanoma than in carcinoma. Thus, the effects of all treatments in combined modality resulted in increased antitumor effectiveness, especially in melanoma, where 81% of melanoma tumors were cured, and resistance to secondary challenge was retained in 59% of the cases. However, a less pronounced effect was observed in carcinoma; tumors were cured in 27% of the cases that had no immune memory, and all tumors grew after secondary challenge.

Taken together, the silencing of MCAM radiosensitizes B16F10 melanoma and TS/A carcinoma, but the effect was more pronounced in melanoma than carcinoma. The combined therapy exerted both antiangiogenic and immune response antitumor effect. However, tumor immunological status is crucial for a sufficient immune system contribution to the overall antitumor effect, including the development of immune memory.

## Figures and Tables

**Table 1:** The antitumor response of B16F10 melanoma after different treatment modalities.

Group	*n*	DT (Days) AM ± SE	GD (Days) AM ± SE	CR		SC (Secondary Challenge)	
				*n*	%	n	%
Control	18	1.2 ± 0.1	0.0 ± 0.1	0	0	0	0
3× EP	12	3.3 ± 0.3	2.1 ± 0.3	0	0	0	0
3× pControl	12	2.1 ± 0.2	0.9 ± 0.2	0	0	0	0
3× pMCAM	12	2.1 ± 0.3	0.9 ± 0.3	0	0	0	0
IR	12	1.7 ± 0.2	0.5 ± 0.2	0	0	0	0
3× GET pControl	16	11.3 ± 2.3	10.1 ± 2.3*	5	31.2	1	20
3× GET pMCAM	14	14.4 ± 2.5	13.2 ± 2.5*	5	35.7	3	60
3× EP + IR	17	6.1 ± 1.0	4.9 ± 1.0	2	11.8	0	0
pControl + IR	16	2.9 ± 0.5	1.7 ± 0.5	1	6.3	1	100
pMCAM + IR	16	6.8 ± 1.9	5.6 ± 1.9	2	12.5	2	100
3× GET pControl + IR	18	31.0 ± 5.9	29.8 ± 5.9*	13	72.2	7	53.8
3× GET pMCAM + IR	21	19.7 ± 4.3	18.5 ± 4.3*	17	81.0	8	47.1

AM: arithmetic mean; SE: standard error of arithmetic mean; DT: tumor doubling time; n: number of all mice in the group; GD: tumor growth delay; CR: complete response, tumor-free animal at day 100, % was calculated by dividing n of animals in complete response by n of animals in the corresponding group; SC: mice resistant to secondary challenge; * *p* < 0.05: statistically significant difference compared to groups: control, 3× EP, 3× pControl, 3× pMCAM, IR, pControl + IR.

**Table 2:** The antitumor response of TS/A carcinoma after different treatment modalities.

Group	*n*	DT (Days) AM ± SE	GD (Days) AM ± SE	CR		SC (Secondary Challenge)	
				*n*	%	n	%
Control	16	2.2 ± 0.2	0.0	0	0	0	0
3× EP	11	2.8 ± 0.2	0.6 ± 0.2	0	0	0	0
3× pControl	11	2.9 ± 0.7	0.7 ± 0.7	0	0	0	0
3× pMCAM	9	2.1 ± 0.2	−0.1 ± 0.2	0	0	0	0
IR	16	3.0 ± 0.3	0.8 ± 0.3	0	0	0	0
3× GET pControl	14	6.3 ± 0.9	4.1 ± 0.9	0	0	0	0
3× GET pMCAM	13	11.1 ± 0.8	8.9 ± 0.8	0	0	0	0
3× EP+IR	16	13.9 ± 2.8	11.7 ± 2.8*	3	18.8	0	0
pControl+IR	15	2.8 ± 0.2	0.6 ± 0.2	0	0	0	0
pMCAM+IR	16	3.6 ± 1.4	1.4 ± 1.4	0	0	0	0
3× GET pControl+IR	13	24.4 ± 3.7	22.2 ± 3.7**	1	7.7	0	0
3× GET pMCAM+IR	15	30.9 ± 2.9	28.7 ± 2.9**	4	26.7	0	0

AM: arithmetic mean; SE: standard error of arithmetic mean; DT: tumor doubling time; n: number of all mice in the group; GD: tumor growth delay; CR: complete response, tumor-free animal at day 100, % was calculated by dividing n of animals in complete response by n of animals in the corresponding group; SC: mice resistant to secondary challenge; * *p* < 0.05: statistically significant difference compared to groups: control, 3× EP, 3× pControl, 3× pMCAM, IR, pControl + IR, pMCAM + IR; ** *p* < 0.05- statistically significant difference compared to all other groups except between GET pMCAM + IR and GET pControl + IR.

**Table 3:** Histological properties in B16F10 melanoma after GET pMCAM and single-dose irradiation.

Group	% of Proliferative Cells (AM ± SE)	% of Necrosis (AM ± SE)	%Apoptosis (AM ± SE)	% of Hypoxic Cells (AM ± SE)	Number of Blood Vessels/Field of View (AM ± SE)	% of Immune Cells (AM ± SE)
Control	92.3 ± 0.8	22.2 ± 3.6	10.4 ± 1.1	4.5 ± 0.5	51.8 ± 3.0	6.8 ± 0.6
3× pControl	92.2 ± 0.8	19.5 ± 2.1	12.9 ± 0.9	5.3 ± 07	47.1 ± 4.7	9.0 ± 0.8
3× pMCAM	83.9 ± 1.6	33.3 ± 3.1	9.8 ± 0.9	6.4 ± 0.5	46.5 ± 2.9	7.9 ± 0.7
3× EP	75.6 ± 1.2	17.5 ± 4.2	17.2 ± 1.6	7.5 ± 0.5	39.9 ± 2.6	11.0 ± 1.1
3× GETpControl	72.7 ± 1.7	56.2 ± 4.9	18.6 ± 1.9	15.6 ± 2.0	35.3 ± 4.2	13.6 ± 1.2
3× GET pMCAM	54.4 ± 2.6	59.2 ± 4.9	34.7 ± 2.9	35.4 ± 2.6	18.1 ± 1.7	23.5 ± 1.9
IR	51.8 ± 2.3	18.7 ± 4.0	18.4 ± 1.3	22.9 ± 2.0	30.1 ± 2.3	23.6 ± 1.7
3× pControl+IR	53.8 ± 3.2	30.0 ± 4.3	20.2 ± 1.4	25.3 ± 2.1	24.5 ± 1.8	26.5 ± 1.3
3× pMCAM+IR	49.2 ± 2.3	40.0 ± 2.9	31.6 ± 1.5	33.9 ± 1.7	25.0 ± 2.0	27.0 ± 1.3
3× EP+IR	57.3 ± 2.9	26.7 ± 4.4	32.9 ± 1.4	45.1 ± 2.2	28.9 ± 2.3	29.7 ± 1.6
3× GET pControl+IR	44.3 ± 3.1	59.2 ± 5.2	39.0 ± 1.9	50.2 ± 2.9	18.9 ± 2.1	44.5 ± 2.3
3× GET pMCAM+IR	36.7 ± 2.1*	90.0 ± 1.3*	60.1 ± 2.9*	62.0 ± 2.0*	12.6 ± 0.9*	63.4 ± 2.5*

AM: arithmetic mean; SE: standard error of arithmetic mean; * *p* < 0.05- statistically significant difference compared to groups: control, 3× EP, 3× pControl, 3× pMCAM, IR.

**Table 4:** Histological properties in TS/A carcinoma after GET pMCAM and single-dose irradiation.

Group	% of Proliferative Cells (AM ± SE)	% of Necrosis (AM ± SE)	% Apoptosis (AM ± SE)	% of Hypoxic Cells (AM ± SE)	Number of Blood Vessels/Field of View (AM ± SE)	% of Immune Cells (AM ± SE)
Control	53.1 ± 2.7	11.2 ± 2.9	3.5 ± 0.7	12.3 ± 1.7	43.9 ± 2.5	3.4 ± 0.6
3× pControl	51.4 ± 1.8	11.5 ± 2.5	4.4 ± 0.6	18.8 ± 1.7	38.9 ± 1.8	4.0 ± 0.7
3× pMCAM	53.4 ± 2.1	13.3 ± 2.5	4.2 ± 0.5	18.6 ± 2.3	39.7 ± 3.2	3.2 ± 0.6
3× EP	46.6 ± 2.1	28.8 ± 4.3	5.0 ± 0.6	22.9 ± 1.8	26.7 ± 1.0	3.3 ± 0.6
3× GETpControl	51.2 ± 2.0	21.7 ± 2.1	12.6 ± 1.2	33.5 ± 2.6	20.5 ± 1.7	6.1 ± 0.8
3× GET pMCAM	44.9 ± 1.9	40.0 ± 8.9	9.1 ± 1.1	33.1 ± 1.7	13.9 ± 1.1	7.3 ± 0.8
IR	33.6 ± 2.2	20.3 ± 3.0	4.2 ± 0.6	13.4 ± 1.8	27.0 ± 1.6	6.1 ± 0.9
3× pControl+IR	39.5 ± 1.6	24.2 ± 1.5	6.9 ± 0.9	17.6 ± 1.2	24.7 ± 1.6	5.7 ± 0.8
3× pMCAM+IR	32.9 ± 1.4	23.8 ± 4.0	6.4 ± 0.7	18.3 ± 0.9	24.6 ± 2.3	8.4 ± 1.1
3× EP+IR	30.8 ± 1.5	34.2 ± 3.7	6.5 ± 0.8	31.8 ± 2.7	26.5 ± 1.6	7.6 ± 0.6
3× GET pControl+IR	33.8 ± 1.3	53.0 ± 4.8	16.1 ± 1.4	47.0 ± 1.5	19.1 ± 1.2	8.8 ± 1.0
3× GET pMCAM+IR	25.7 ± 2.1*	64.2 ± 4.0**	27.6 ± 2.9**	54.4 ± 2.1**	11.0 ± 1.2**	21.9 ± 2.1**

AM: arithmetic mean; SE: standard error of arithmetic mean; EP: application of electric pulses; GET: gene electrotransfer; IR: single-dose irradiation, 15 Gy; pMCAM: intratumorally injection of plasmid DNA-encoding shRNA for MCAM; pControl: intratumorally injection of control plasmid; * *p* < 0.05: statistically significant difference compared to groups: control, 3× EP, 3× pControl, 3× pMCAM; ** *p* < 0.05: statistically significant difference compared to groups: control, 3× EP, 3× pControl, 3× pMCAM, IR.

## Supplementary Materials

The following are available online at https://www.mdpi.com/2076-393X/8/1/135/s1, Table S1: Radiosensitization of B16F10 melanoma and TS/A carcinoma cells after MCAM silencing.
