# Supplementary material for: Intratumoral Gene Electrotransfer of Plasmid DNA Encoding shRNA against Melanoma Cell Adhesion Molecule Radiosensitizes Tumors by Antivascular Effects and Activation of an Immune Response
Source: Vaccines (Basel). 2020 Mar 19;8(1):135. doi: 10.3390/vaccines8010135 (PMC7157247; doi:10.3390/vaccines8010135)
Supplement: Supplementary file 1 [file vaccines-08-00135-s001.pdf]

vaccines

*Supplementary Material*

**Table 1.** Radiosensitization of B16F10 melanoma and TS/A carcinoma cells after MCAM silencing.

| | B16F10 cells | | TS/A cells | |
|---|---|---|---|---|
| **Group** | **$IC_{90}$ (Gy)** | **EF** | **$IC_{90}$ (Gy)** | **EF** |
| **IR** | 6.11 | | 7.78 | |
| **pControl+IR** | 5.09 | 1.20 | 5.78 | 1.35 |
| **pMCAM+IR** | 4.96 | 1.23 | 5.77 | 1.35 |
| **ER+IR** | 4.93 | 1.24 | 6.42 | 1.21 |
| **GET pControl+IR** | 4.71 | 1.30 | 4.74 | 1.64 |
| **GET pMCAM+IR** | 3.89 | 1.57 | 4.53 | 1.72 |

$IC_{90}$- a dose of irradiation (IR) that reduced survival of cells to 10%; EF- enhancement factor calculated by dividing of $IC_{90}$ value of IR group with a group of combined treatments with IR.
